# Microbiological evaluation of different hand drying methods for removing bacteria from washed hands

**DOI:** 10.1038/s41598-019-50239-4

**Published:** 2019-09-24

**Authors:** Lorna K. P. Suen, Vanessa Y. T. Lung, Maureen V. Boost, Cypher H. Au-Yeung, Gilman K. H. Siu

**Affiliations:** 1 Squina International Centre for Infection Control, School of Nursing, The Hong Kong Polytechnic University, Kowloon, Hong Kong; 2 School of Optometry, The Hong Kong Polytechnic University, Kowloon, Hong Kong; 3 Department of Health Technology and Informatics, The Hong Kong Polytechnic University, Kowloon, Hong Kong

**Keywords:** Applied microbiology, Risk factors

## Abstract

Proper drying of hands after washing is an integral part of hand hygiene. An experimental study on 30 subjects using multiple comparisons of six hand drying methods including 1) drying on own clothes, 2) drying with one paper towel, 3) drying with two paper towels, 4) drying with a warm air dryer while holding hands stationary for 20 s, 5) drying with a warm air dryer while hand rubbing for 20 s, and 6) drying with a jet air dryer until complete dryness was achieved. It aimed to determine the effectiveness of different hand drying methods for removing bacteria from washed hands, so as to identify the optimum method using minimum resources. Our study demonstrated that the use of jet air dryers is the best method to eliminate bacteria on hands, whereas drying hands on one’s own clothes is the least effective. Drying hands in a stationary position could remove more bacteria than rubbing hands when using a warm air dryer for 20 s, which mimics people’s usual hand-drying practice. No significant difference in bacteria reduction was detected between the use of one or two paper towels for hand drying; therefore, using fewer resources is recommended to maintain environmental sustainability.

## Introduction

The importance of thorough cleansing of the hands with soap and water or a hand sanitiser is well documented^1–3^. Hands which are inadequately dried are more likely to transmit microorganisms than those which are thoroughly dried^4^. However, few studies have evaluated the effectiveness of different drying methods to remove microorganisms from the hands and the reported results are inconsistent.

Redway and Fawdar^5^ assessed changes in the number of bacteria on the hands before and after the use of various hand drying methods. The authors concluded that use of paper towels could reduce the numbers of all types of bacteria on the hands, whereas the increase in numbers of bacteria was relatively lower with the jet air dryer than with the hot air dryer. In contrast, Gustafson *et al*.^6^ adopted a modified glove-juice sampling procedure and detected no significant difference in bacteria removal efficiency among hand-drying methods, including paper towels, cloth towels, hot air dryers, and spontaneous evaporation. Even though the glove-juice sampling procedure permits the sampling of interdigital areas and provides comprehensive sampling of skin bacteria, this method could not reflect the bacterial numbers remaining on different hand regions. Ansari *et al*.^7^ mentioned that friction is often applied when the hands are dried with paper or cloth towels, but did not incorporate any friction in hand drying because of the difficulties in standardising the procedures under field conditions. Whether friction can further reduce contamination during hand drying remains to be determined.

The drying efficiency of individual drying methods also varies. Hot air dryers are much slower than other methods, such as paper towels, taking around 45 s to reduce residual water to 3%^4^. The significantly poorer hygiene performance of hot air dryers compared with other drying methods could be due to their low drying efficiency and, consequently, the greater amount of water remaining on the hands. An epidemiological investigation of the general public in Hong Kong^8^ found that over 40% of the 815 respondents always/sometimes rubbed their hands on their own clothing as a means for hand drying, and over 70% of the respondents spent less than 20 s when using hand dryers.

The use of paper towels may have adverse effects relating to waste disposal and environmental sustainability. Joseph *et al*.^9^ attempted to conduct a comparative life cycle assessment of conventional hand dryers and roll paper towels as hand drying methods. They found that using a conventional hand dryer had a lower environmental impact than using two paper towels from a roll dispenser. Therefore, whether the use of one paper towel could achieve comparable drying effects to the use of two paper towels is worth exploring.

Considering the inconsistent findings of previous studies and the knowledge gap identified in the literature, further studies using a scientific approach are warranted to determine the effectiveness of different hand drying methods for removing bacteria from washed hands, in order to identify the optimum hand drying method using minimum resources.

## Methodology

This is an experimental study of one group using multiple comparisons, aimed to evaluate the differences in the reduction of colony-forming units (CFU) on the hands before and after using different hand-drying methods. These methods included: 1) drying on own clothes, 2) drying with one paper towel, 3) drying with two paper towels, 4) drying with a warm air dryer while holding hands stationary for 20 s, 5) drying with a warm air dryer while hand rubbing for 20 s, and 6) drying with a jet air dryer until complete dryness. The use of 20 s when using a warm air dryer aimed to reproduce the usual hand-drying practice of the population when using this device^8^.

### Study participants

Potential participants who aged 18 or above who agreed to participate in this study were recruited. Any having acute or chronic nail or skin disorders/lesions, including eczema, or fever were excluded. For all six hand drying methods tested, the change in bacterial count between pre-washing and after drying reported in previous studies^6,10,11^ was used as a reference to determine sample size. A sample size of 30 participants was calculated as adequate to detect the mean difference with an effect size of 0.68, with 90% power, and 5% level of significance.

### Preparation of *Serratia marcescens* culture

Bartzokas *et al*.^12^ suggested the use of *S*. *marcescens* to study pathogen transmission on the hands. It was adopted in this study as the marker organism because of its production of pink or red pigment. This rod-shaped Gram-negative bacteria is a biosafety-level-one organism, indicating its safety to humans, except for those who are immunosuppressed. Before the experiment, *S*. *marcescens* strain ATCC 13880 was cultured for 3 days to produce an artificial contamination source and was further cultured to a suitable concentration.

### Devices for the six hand drying methods

The paper towels (Vinda M-Fold Hand Towel) to be used for hand drying, each measuring 220 mm × 230 mm, were sterilised in an autoclave for 15 min before use. The warm air dryer (KDK T09BC), which was newly installed for the research, has a velocity of 100 m/s and 1020 W power. Washed hands were held 10 cm from the nozzle of the air dryer during use, and either rubbed or held stationary for 20 s to mimic usual hand-drying practice. The jet air dryer (Dyson Airblade dB), which has a velocity of 192 m/s and 1600 W power was also newly installed^13^. Participants placed their hands into the dryer in a standardised manner for 10 s when using; specifically, they held their hands still, with fingers pointing downwards and slightly spread out until thorough dryness was achieved.

### Procedures

The experiments were conducted at the ‘Hand Hygiene Skill Station’ of the university with a mean temperature of 23 °C and a humidity of 55%. Ethical approval was obtained from the Human Research Ethics Review Committee of the Hong Kong Polytechnic University (Reference number: HSEARS20170516001) who approved all the experimental protocols adopted in this study. All methods being used were carried out in accordance with relevant guidelines and regulations. Participation in the study was voluntary. Written informed consent was obtained from each subject following explanation of the risks and benefits of their participation.

Demographic data (gender, age, educational level, and side of dominant hand) and information about hand drying habits were collected. To eliminate any confounding effect due to test order or residual bacteria on the hands, the order participants performed the six hand-drying methods was randomly assigned by a computer-generated table. The hands of each participant were dried by these testing methods guided by this random sequence. The standardised procedures adopted of the experiment were as follows:

1. Hands were washed with Funchem liquid hand soap (NL-500C) for 20 s following the handwashing guidelines recommended by the Centers of Disease Control and Prevention^3^. Funchem was chosen as it is one of the commonly used hand soaps in the hospitals of Hong Kong. The active ingredients of this unmedicated hand soap mainly include surfactants, dimethylhydantoin, and glycerine. For consistency, a full shot of hand soap (~2 mL) was squeezed from the dispenser. The handwashing procedure involved rubbing the lathered hands together vigorously for 20 s, covering all surfaces of the hands and then rinsing hands thoroughly for 20 s under running water.
2. *S*. *marcescens* was cultured in a tryptone soya broth for 48 h to reach a concentration of 10^9^ CFU/ml for use within 6 h. The hands were artificially contaminated with gauze soaked in six aliquots of *S*. *marcescens* bacterial suspension (20 mL each, 120 mL in total) for 15 s. Excess solutions were allowed to drip away during contamination.
3. A pre-washing sample on the hand was taken. For consistency purposes, the samples were taken on the dominant hand of the participant. The finger and palm regions within an area of 5 × 5 cm^2^ were individually sampled. A sterile cotton swab moistened with sterile 0.9% saline was firmly applied for 15 times horizontally and 15 times vertically in a zigzag pattern^14,15^ and rotated while sampling.
4. The hands were washed again with one full shot of Funchem liquid hand soap for 20 s and rinsed well under running water^3^. The hands were shaken dry at least five times to remove excess water before drying.
5. The hands were dried according to one of the six hand drying methods in random sequence.
6. A post-drying sample was taken, using the procedure described in step 3.
7. The procedures of steps (1) to (6) were repeated for the other hand-drying methods under testing. A 15 min break was provided between each testing method.
8. The swabs were transferred into 1 mL 0.9% normal saline, and 0.2 mL of the solution was pipetted to the 1% glucose-supplemented nutrient agar plate in Petri dishes for culture. All cultures were incubated at 37 °C for 24 h.
9. After incubation, the CFUs of the marked organism were counted automatically, and photographed using a BIOMIC (Giles Scientific, USA) laboratory plate reader, and the plates were disposed as biohazard materials in accordance with campus regulations after use.
10. Upon completion of the experiment, a shopping coupon of HK$50 (~₤5), a bottle of hand cream, and a hand wax treatment were provided to the participants as a token of appreciation. An individual report of the experiments was also given to the participants for reference.

### Data analysis

Descriptive statistics was conducted for the demographics and computation of *S*. *marcescens* values. For the six drying methods, the experiment focused on the difference in *S*. *marcescens* counts, which was identified as the changes between prewashed and post-dried hands. Wilcoxon signed-rank test was used to determine the within-group comparisons of *S*. *marcescens* counts before and after each hand drying method, the bacteria removal rate of using warm air dryer with holding hands stationary versus hand rubbing and using one paper towel versus two paper towels for hand drying. The Friedman test was used to determine the mean rank of bacteria reduction using different hand drying methods. Analyses were conducted using SPSS (version 25.0). Statistical significance was considered at *p < *0.05.

## Results

### Sociodemographic characteristics and hand drying behaviours of respondents

Data were collected from June to November 2017. A total of 30 participants (9 males and 21 females) were recruited. Most participants were in their twenties, with tertiary or above education, and were right handed.

With respect to hand drying behaviours, more than one-third of participants (*n* = 12, 40%) admitted that they always/sometimes dry their hands on their own clothing. The respondents generally preferred using paper towels supplied by washrooms (*n* = 29, 96.7%) and limit the use of paper towels to two pieces (*n* = 27, 93.2%). Over half of the respondents rubbed their hands when using a warm hand dryer (*n* = 15, 51.7%). The average time for using warm hand dryers was generally inadequate, with over 60% of respondents taking ≤10 s when using a warm (*n* = 18, 62.1%) or jet hand dryer (*n* = 19, 67.8%) (Table 1).

**Table 1:** Socio-demographic characteristics and hand drying behaviour of respondents (*n* = 30).

Variables	n (%)※
**Age group**	
20–29	27 (90.0)
30–39	1 (3.3)
40–49	0 (0.0)
50–59	2 (6.7)
**Gender**	
Male	9 (30.0)
Female	21 (70.0)
**Educational level**	
Secondary	2 (6.7)
Tertiary/College or above	28 (93.3%)
**Dominant hand**	
Left	4 (13.3)
Right	26 (86.7)
**Rub hands on own clothing**	
Always	2 (6.7)
Sometimes	10 (33.3%)
Never	18 (60.0)
**Paper towels supplied by the washroom**	
Always	22 (73.4)
Sometimes	7 (23.3)
Never	1 (3.3)
**Warm hand dryer**	
Always	6 (20.0)
Sometimes	19 (63.3)
never	5 (16.7)
**Jet hand dryer**	
Always	6 (20.0)
Sometimes	18 (60.0)
Never	6 (20.0)
**How many paper towels do you commonly used**	
One	13 (44.8)
Two	14 (48.4)
Three	1 (3.4)
Four or more	1 (3.4)
Not applicable	1
**If warm hand dryer is used**, **how do you usually position your hands?**	
Rubbing hands during drying	15 (51.7)
Hold hands stationary during drying	14 (48.3)
Not applicable	1
**Average time for using warm hand dryer** (**in seconds**)	
Less than 5 sec	2 (6.9)
5–10 sec	16 (55.2)
11–20 sec	9 (31.0)
21–30 sec	2 (6.9)
31–40 sec	0 (0.0)
41 sec or more	0 (0.0)
Not applicable	1
**Average time for using jet hand dryer** (**in seconds**)	
Less than 5 sec	3 (10.7)
5–10 sec	16 (57.1)
11–20 sec	8 (28.6)
21–30 sec	1 (3.6)
31–40 sec	0 (0.0)
41 sec or more	0 (0.0)
Not applicable	2

^※^Not applicable cases were excluded from percentage calculation and the analyses. applicable cases were excluded from percentage calculation and the analyses.

### Results of hand drying experiments

Apart from the pre-determined duration of 20 s for using the warm air dryers, the average duration used by the participants for drying on own clothes, drying by one paper towel, and drying by two paper towels were 16.7 s (5 to 31), 17.5 s (8 to 32), and 20.6 s (10 to 57) respectively. The average duration to achieve complete dryness of the hands when using the jet air dryer was 27.4 s (range 15 to 42). All samples were taken from the dominant hand of the participants. The *S*. *marcescens* counts on both the palm and finger regions were significantly reduced (*p* < 0.001) before and after using each hand drying method. Drying hands on own clothes resulted in significantly greater bacterial reduction on the fingers than on the palms (*p* = 0.028), whereas no differences in these regions could be detected using other hand drying methods (Table 2).

**Table 2:** The change in *S*. *marcescens* counts on different regions of hands before and after using various hand drying methods.

Hand drying methods	Mean (SD) of *S*. *marcescens* counts/cm^2^						Mean bacteria reduction on hands		
	Palm			Fingers					
	Baseline	Post	*p*-value #	Baseline	Post	*p*-value #	Palm (%)	Fingers (%)	Z value (*p*-value)
On own clothes	212.0 (95.8)	68.1 (166.1)	***	224.7 (135.8)	33.2 (42.6)	***	26.5	81.6	−2.19 (0.028)*
By one paper towel	232.9 (112.6)	11.2 (15.5)	***	218.1 (130.38)	11.81	***	93.6	87.3	−0.85 (0.393)
By two paper towels	234.9 (116.9)	18.03 (49.0)	***	198.4 (103.0)	13.2 (15.1)	***	92.7	91.5	−1.12 (0.262)
By warm dryer (hands in rubbing motion for 20 s)	259.1 (153,5)	17.5 (20.2)	***	208.8 (115.2)	19.2 (20.8)	***	90.0	88.1	−0.87 (0.382)
By warm dryer (hands held stationary for 20 s)	238.8 (142.6)	9.3 (11.3)	***	236.6 (96.1)	12.9 (17.9)	***	93.3	93.3	−1.41 (0.159)
By jet air dryer (until dry)	205.4 (92.5)	11.9 (24.5)	***	200.4 (109.3)	6.2 (6.2)	***	91.9	95.9	−0.29 (0.770)

^#^Wilcoxon signed ranked test.

*Statistically significant at *p* < 0.05.

**Statistically significant at *p* < 0.01.

***Statistically significant at *p* < 0.001.

The results of the Friedman test indicated a statistically significant difference in the reduction of bacteria among the six hand drying methods (X^2^ = 19.22, df = 5, *p* = 0.002). Use of the jet air dryer was the most effective method, having the best performance in removing *S*. *marcescens*, with a mean rank of 4.32 and a removal percentage of 94.9%. Drying on one’s own clothes was the least effective method, with a mean rank of 2.63 and a removal percentage of 59.7. One participant, who was found to have a six-fold increase in their *S*. *marcescens* count after drying the hands on his clothes, admitted that the clothes he used to dry his hands had been worn the day before (Table 3).

**Table 3:** The mean rank of total bacteria reduction using different hand drying methods.

Hand drying methods	Mean rank	Mean bacteria reduction on hands (%)	Maximum (%)	Minimum (%)
On own clothes	2.63	59.7	6-fold increase	98.7
By one paper towel	3.80	94.0	71.1	99.9
By two paper towels	3.15	92.3	49.6	100.0
By warm dryer (hands in rubbing motion for 20 s)	3.00	90.2	60.4	99.6
By warm dryer (hands held stationary for 20 s)	4.10	93.5	65.1	100.0
By jet air dryer (until dry)	4.32	94.9	57.8	100.0
Friedman test (test statistics)	X^2^ = 19.22, df = 5, *p* = 0.002			

The bacteria removal rate of using a warm air dryer was significantly higher with holding hands stationary than with hand rubbing (z = −2.19, *p* = 0.028). No significant difference in bacteria removal rate was found between using one paper towel or two paper towels for hand drying (z = −1.64, *p* = 0.102).

## Discussion

This study demonstrated that using a jet air dryer for hand drying is the best method to eliminate bacteria on the hands, whereas drying hands on one’s own clothes is the least effective. Use of the jet air dryer was superior to other hand drying methods in this study, possibly because complete dryness of the hands was achieved with an average duration of 27.4 s, which was, however, much longer than the manufacturer’s claim of 10 s^16^. In comparison, the participants only used the warm air dryer for 20 s, in order to mimic usual reported hand-drying practice, but this duration was not adequate to achieve thorough hand dryness. Patrick *et al*.^4^ reported that hot air dryers require 45 s to reduce the residual water to 3%. Hands, which are inadequately dried, are more likely to transmit microorganisms than those which are completely dried^4^. Jet air hand dryers have an advantage of requiring a relatively shorter drying time than warm air dyers, which can help enhance compliance for achieving complete dryness. The duration reported by the participants when using the jet air dryer in daily life was much shorter than the average duration they performed in this trial. Therefore, whether the use of jet air dryers is actually superior to other hand drying methods under real-life circumstances has yet to be determined.

Drying hands on clothes can compromise the benefits of handwashing^17^. The situation may worsen hand hygiene, especially when the clothes are dirty, because the clothing itself could be a source of contamination and compromise the effect of hand washing. Gram-positive and Gram-negative bacteria can survive on the surface of clothes for approximately 4 h and sometimes up to 24 h^18^. This study showed that drying hands on one’s own clothes has significantly greater bacteria reduction on fingers than on palms, probably because people tend only to dry their fingers on their clothes, rather than the whole palm. Around 40% of the participants indicated that they always/sometimes dry their hands on their own clothing. Therefore, the general public should be educated to avoid drying their hands on dirty clothing.

By evaluating the cleaning effect of friction applied during hand drying, this study showed that drying hands in a stationary position could remove more bacteria than rubbing hands throughout the drying process under the same conditions (*p* < 0.05). The possibility of bacteria transfer from dryers onto the hands was minimal, because the devices were newly installed for research purposes. Yamamoto *et al*.^11^ used a contact-plate method to compare the differences between when the hands were rubbed and when the hands were held stationary, when a warm air dryer was used. Bacteria on rubbed hands increased significantly, whereas those on hands held stationary decreased, which agrees with the findings of the current study. The rubbing process may tend to draw out commensal bacteria to the skin surface from deep inside the pores and under the fingernails. In addition, the area of the hands that can be exposed to warm air for evaporation is decreased while rubbing as compared with holding hands stationary, thus affecting drying efficiency. The water residue remains on the palm and fingers and did not evaporate when our participants rubbed their hands. After 20 s of drying, almost all participants found that their hands were still wet and had not reached dryness. Our study demonstrated that the bacteria removal power of the warm air dryer was affected by how the hands were positioned and the dryness of the hands after using the devices. So far, little research has been conducted to investigate this issue. Therefore, additional scientific evidence should be generated in future studies to determine the causal relationship between performing friction of the hands and the amount of bacteria remaining on the hands.

Although jet air dryers were shown to be superior in removing bacteria, in terms of the effect on cross-contamination, jet air and warm air dryers increase bacterial aerosolisation from the hands. The dispersion of bacteria by warm air dryers has been found within a radius of approximately 3 ft. from the device to the surrounding environment, whilst no dispersal of bacteria occurred when paper towels were used^19^. In a separate study, Kimmitt and Redway^20^ attempted to use an MS2 bacteriophage model to compare different hand-drying methods (paper towels, warm air dryers, and jet air dryers) for their potential to disperse microbes and contaminate the environment during use. The authors concluded that jet air dryers, due to the powerful velocity with which they emit air during use, disperse an average of >20- and >190-fold more plaque-forming units than warm air dryers and paper towels, respectively, at all distances tested up to 3 m. Other studies, using Lactobacilli contamination^21^ or examining skin flora contamination^22^ have confirmed this dispersion effect. Thus, dryers may be unsuitable for use in healthcare settings or the food industry because of possible microbial cross-contamination via airborne dissemination to the environment or other bathroom users^21^. Therefore, future studies should evaluate the spatial distribution of bioaerosols released during the operation of different types of hand drying devices and determine whether the design of these dryers can be adapted to reduce dispersion.

A review of 12 studies^23^ found little agreement regarding the relative effectiveness of electric air dryers. However, most studies suggest that paper towels can dry hands efficiently, remove bacteria effectively, and cause less contamination of the washroom environment than the use of hand dryers. Previous reports^5,24,25^ have shown that paper towels are more effective in removing bacteria on the fingertips than on palms, but this situation was not observed in our study. In contrast to earlier studies, a recent experimental study^26^ reported that drying hands with paper towels increased the number of bacteria, including potentially pathogenic species, on skin compared to a jet air dryer. It is possible that the potential microbial hazards were present in recycled paper towels. Gendron *et al*.^27^ attempted to investigate the bacterial load on six different brands of unused paper towels and was able to culture between 10^2^ and 10^5^ colony-forming units (CFU) of bacteria per gram of paper towel. They also demonstrated that such contaminating bacteria could be transferred from the paper towels to the hands of users during hand-drying. It is worth noting that, for the purpose of the study, the paper towels used in our study were sterilised before use, and this might have implications on the findings.

From the perspective of environmental sustainability, Joseph *et al*.^9^ reported that using paper towels negatively impacts the environment, because their consistency makes them hard to decompose. In the present study, no significant difference in bacteria reduction was detected between the use of one paper towel and two paper towels for hand drying. Therefore, using fewer resources is recommended to improve environmental sustainability.

### Limitations and recommendations

This study has some limitations. The participants only used the warm air dryer for 20 s, which is inadequate to achieve thorough hand dryness; thus, the results may have differed if the participants were allowed to use the device until complete hand dryness was achieved. The short interval of 15 min between each testing method could have created bias in the results due to the residual effects of the hand soap on the bacterial count. Even though the use of *S*. *marcescens* serves as a safe and ideal contamination agent, as it could produce a pink or red pigment for easy identification in hand hygiene studies, information about the bacterial species which may be present on dirty clothing was lacking^28^. The participants in the study were mostly younger females, with higher education levels. Being female, middle aged and tertiary education level have been reported as protective factors for improved hand hygiene knowledge, and these factors possibly affect hand hygiene behaviours^8^. A more representative sample in terms of socio-demographic distribution should be considered in future studies. Other research directions, including the evaluation of the spatial distribution of bioaerosols released during the operation of different types of hand drying devices, measures to increase hand hygiene adherence, the cost analysis, and environmental effect of different hand drying methods, should be further investigated.

## Conclusion

The findings of this study demonstrated that the use of jet air dryers are most effective in eliminating bacteria on the hands, whilst drying hands on one’s own clothes is the least. Drying hands in a stationary position could remove more bacteria than rubbing hands when using a warm air dryer for 20 s, which replicates people’s usual hand-drying practice. Overall reluctance to spend sufficient time under a conventional dryer means that hands will remain wet allowing better survival for organisms. As rubbing reduces even the limited efficiency of warm air dryers, users should be instructed to hold hands stationary during use. No significance difference in bacteria reduction was detected between use of one or two paper towels for hand drying; therefore, using fewer resources is recommended to maintain environmental sustainability. As there may be cross-contamination risks associated with use of jet air dryers, hand drying in hospital and other high risk environments should be performed using a paper towel

## Data Availability

The datasets used and/or analysed during the current study are available from the corresponding author.
